# Psychological Effects of Heart Rate and Physical Vibration on the Operation of Construction Machines: Experimental Study

**DOI:** 10.2196/31637

**Published:** 2021-09-15

**Authors:** Nobuki Hashiguchi, Jianfei Cao, Yeongjoo Lim, Shinichi Kuroishi, Yasuhiro Miyazaki, Shigeo Kitahara, Shintaro Sengoku, Katsushi Matsubayashi, Kota Kodama

**Affiliations:** 1 Graduate School of Technology Management Ritsumeikan University Ibaraki Japan; 2 Faculty of Business Administration Ritsumeikan University Ibaraki Japan; 3 Metropolitan Area Branch Civil Engineering Department Kumagai Gumi Co, Ltd Shinjuku-ku Japan; 4 Civil Engineering Business Headquarters Kumagai Gumi Co, Ltd Shinjuku-ku Japan; 5 School of Environment and Society Tokyo Institute of Technology Minato-ku Japan; 6 National Institute of Technology Tokyo College Tokyo Japan

**Keywords:** heart rate variability, complexity, vital signs, vibration at work, stress, wearable technology, remote operation, monitoring

## Abstract

**Background:**

A construction method has emerged in which a camera is installed around a construction machine, and the operator remotely controls the machine while synchronizing the vibration of the machine with the images seen from the operator's seat using virtual reality (VR) technology. Indices related to changes in heart rate (HR) and physical vibration, such as heart rate variability (HRV) and multiscale entropy (MSE), can then be measured among the operators. As these indices are quantitative measures of autonomic regulation in the cardiovascular system, they can provide a useful means of assessing operational stress.

**Objective:**

In this study, we aimed to evaluate changes in HR and body vibration of machine operators and investigate appropriate methods of machine operation while considering the psychological load.

**Methods:**

We enrolled 9 remote operators (18-50 years old) in the experiment, which involved 42 measurements. A construction machine was driven on a test course simulating a construction site, and three patterns of operation—riding operation, remote operation using monitor images, and VR operation combining monitor images and machine vibration—were compared. The heartbeat, body vibration, and driving time of the participants were measured using sensing wear made of a woven film-like conductive material and a three-axis acceleration measurement device (WHS-2). We used HRV analysis in the time and frequency domains, MSE analysis as a measure of the complexity of heart rate changes, and the ISO (International Standards Organization) 2631 vibration index. Multiple regression analysis was conducted to model the relationship among the low frequency (LF)/high frequency (HF) HRV, MSE, vibration index, and driving time of construction equipment. Efficiency in driving time was investigated with a focus on stress reduction.

**Results:**

Multiple comparisons conducted via the Bonferroni test and Kruskal-Wallis test showed statistically significant differences (*P*=.05) in HRV-LF/HF, the vibration index, weighted acceleration, motion sickness dose value (MSDV_z_), and the driving time among the three operation patterns. The riding operation was found to reduce the driving time of the machine, but the operation stress was the highest in this case; operation based on the monitor image was found to have the lowest operation stress but the longest operation time. Multiple regression analysis showed that the explanatory variables (LH/HF), RR interval, and vibration index (MSDV_z_ by vertical oscillation at 0.5-5 Hz) had a negative effect on the driving time (adjusted coefficient of determination R^2^=0.449).

**Conclusions:**

A new method was developed to calculate the appropriate operating time by considering operational stress and suppressing the physical vibration within an acceptable range. By focusing on the relationship between psychological load and physical vibration, which has not been explored in previous studies, the relationship of these variables with the driving time of construction machines was clarified.

## Introduction

### Background

Construction work in Japan, which is often affected by natural disasters such as large-scale earthquakes, windstorms, floods, and volcanic disasters, has been attracting attention for initiating the remote operation of construction machines from a safe location in cases where the actual location faces a risk of secondary disasters such as mudslides [1,2]. Compared to actual machine operation, remote operation requires care and consideration because it is difficult to ascertain the situation of the construction machine and the working environment; this tends to place a higher psychological load on the operator. In this construction method, the operator recognizes the actual situation of the construction machine (inclination and shaking) through a monitor image and the vibration of the construction machine. The key challenge is to operate the machine reliably and efficiently without increasing the psychological load of the operation. Very few reports on typical construction focus on the psychological load because of the high priority given to avoiding physical hazards for workers [3]. Therefore, this study focused on the psychological load and stress experienced by technicians operating construction equipment to determine efficient and appropriate operations.

Stress resulting from physical and psychological loads in any job can reduce job efficiency by decreasing the sense of satisfaction and well-being. When analyzing stress, observing variations in the heartbeat interval provides a quantitative measure of the autonomic regulation of the cardiovascular system in response to stressors [4,5]. Regarding psychological workload, workers need to be consistently aware of many things, and an imbalance in the resources contributing to psychological workload could pose a safety risk. The assessment of psychological load has gained precedence in many job tasks [6]. Therefore, the relationship of heart rate variability (HRV) and multiscale entropy (MSE) with psychological workload has been investigated in several studies [6-9].

Recent advances in wearable technology have provided an opportunity to easily monitor the biometric information and physical condition of the subjects. The use of wearable devices to monitor autonomic nervous system activity through heart rate (HR) observations is economical, with easy access to data [10,11]. Evaluation of daily changes in HR provides useful information for understanding heart health status with respect to workloads [12], mental states [13,14], and physical conditions [15]. Previous studies have reported that a 5-minute HRV measurement provides a highly accurate analysis [5].

### Life Events and Stress

Stress can be attributed to multiple factors, such as physical, chemical, and biological stressors. Holmes and Rahe [16] have pointed out the psychological and social stressors in social life. Factors that contribute to these stressors include relationships, family problems, and occupational problems. Lazarus and Cohen [17] also argued that the daily hustle of life, comprising minor daily irritations, contributes more to the negative effects on our physical and mental health than the less frequent serious life events. Psychological and social stressors are complex and diversify annually with the changes in our environment and social conditions.

When the human body encounters an unpleasant or harmful event, a defensive reaction of the body and mind occurs. The level of arousal increases to alert us to the outside world, and anxious feelings emerge. In the body, the autonomic nervous system, called the sympathetic nervous system, and the endocrine system, which secretes adrenal cortical and other hormones, becomes more active [5,18]. Through experiments on animals, Hance Selye [19] revealed that in contrast to the usual level of resistance to stress, the warning response phase includes a shock phase in which resistance decreases immediately after encountering the stressor, and then shifts to an antishock phase in which resistance increases. The defensive response of the body and mind after encountering a stressor changes significantly over time. The physical activity and resistance of individuals drop significantly below their usual levels during the stress phase. Then, in the antishock phase, adrenaline is secreted, and the sympathetic nervous system becomes more active, resulting in higher levels of arousal and activity [20]. The liver produces glucose to supply the whole body with energy for activity, and the bronchi tend to become thicker, and respiration becomes faster to take in more oxygen. Fluctuations such as an increased HR occur to pump large amounts of nutrients and oxygenated blood throughout the body [21].

### Research Objectives

In this study, we aimed to investigate the effects of stress and body vibrations on HR and consider machine operation that accounts for the load caused by work vibration during riding and remote operation based on the characteristics of HR information.

The results of this analysis are expected to lead to a new computational model for evaluating operation stress and driving time according to the widely adopted sensing wear and vital signs collected using HR sensors and three-axis accelerometers.

## Methods

### Measurement Tools

Multimedia Appendix 1 lists the measurement devices and infrastructure considered in this study. We measured the HR and physical activity of machine operators on the basis of the electrocardiogram (ECG) signals captured using the sensing wear worn by the operators. Sensing wear is an underwear-type shirt fitted with a biometric information sensor (for detecting HR). As sensing wear clothing is made of stretchable fabric, stretchable ECG electrodes were integrated with the hardware for measuring the HR. The HR was detected using the RR intervals (RRIs) in the ECG signals. The RRI and body acceleration extracted from the ECGs were measured to evaluate the load of the operator in the work environment. The devices used for physiological measurements were WHS-2 for HR measurements and three-axis accelerometers for measuring vital signs (Union Tool Co Ltd), COCOMI (Toyobo Co Ltd) as the sensing wear, and a CC2650 data acquisition device (Texas Instruments).

**Figure 1:**
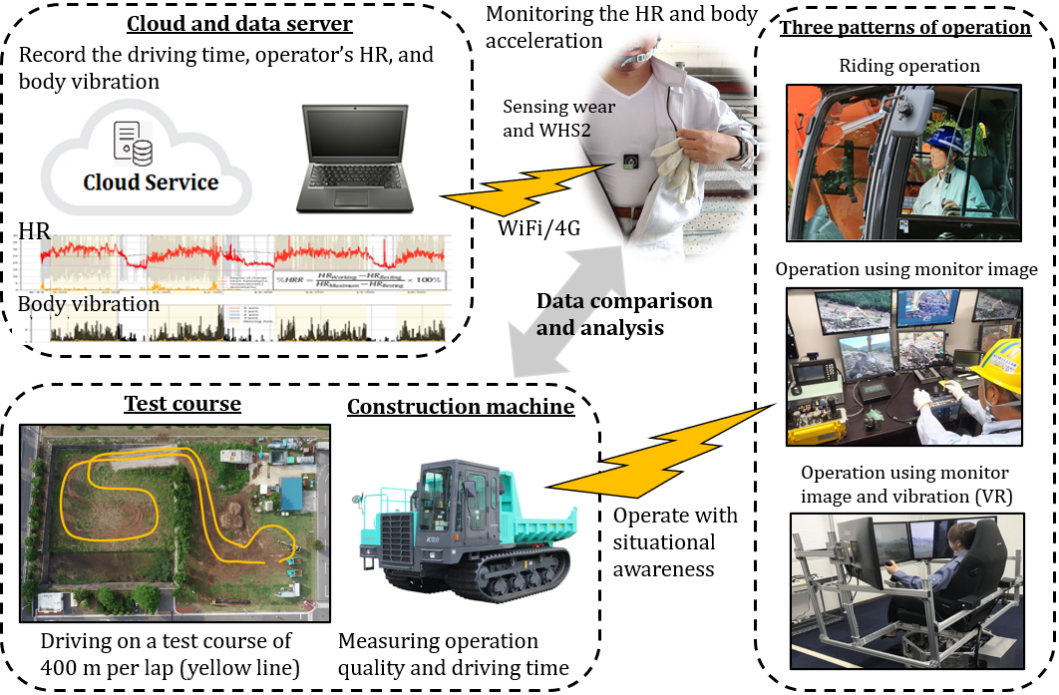
Schematic representation of the monitoring platform. HR: heart rate; VR: virtual reality.

Using a Bluetooth low-energy device, the HR and three-axis acceleration data were sent to the data acquisition device used by the operators. Subsequently, the data from the acquisition device were transmitted to and stored on the cloud server installed on the network using the established wireless access point (using transfer devices based on WiFi and 4G) in the work area. The measurement device and system configuration used in this study are shown in Figure 1.

### Participants

The data were collected at the Tsukuba Technical Research Institute of Kumagai Corporation (Kumagai-gumi, Inc, Chiyoda-ku, Tokyo), a construction company, on July 29 and 30, 2020, and February 18, 2021. A construction company employee (who is also a member of our research team) recruited 20 operators within the company, and engineers who responded to the call participated in this experiment. Participants were included if they were healthy adults aged 18-50 years and excluded if they had any neurological or cardiovascular diseases. As the operators participating in this experiment had highly specialized knowledge and skills and were busy with their daily work, it was difficult for many applicants to participate in the experiment. Hence, 9 construction technicians trained in remote control were selected from construction companies (age: 35.6, SD 12.8 years; height: 168.7, SD 4.1 cm; weight: 71.1, SD 13.2 kg; BMI: 24.9, SD 4.4). Participants drove a crawler carrier (IC120-2, KATO WORKS Co, Ltd) while wearing a device on a test course that mimicked a construction site with a 400-meter lap. All participants were familiar with the experimental procedures.

### Protocol

Our research group investigated the potential risks and discomfort of the participants as well as the privacy issues relating to data collection prior to commencing data collection. The sensing garment was confirmed to be a noninvasive device that does not interfere with machine operation. In accordance with the Declaration of Helsinki, the human genome, and the Universal Declaration of Human Rights, the protocol for data collection was approved by the Ritsumeikan University’s Research Ethics Review Board (number BKC-2019-038). In addition, an explanation of the participants' rights was included in the informed consent form distributed to all participants before data collection, ensuring the confidentiality of the participants' data. Employee names were not used in the experiments and data analysis to minimize the risk of disclosure of personal information. Instead, a personal identification code (identifier) was assigned to each participant.

### Data Collection and Analysis

The SPSS Version 26 for Windows (IBM Corp) and Excel add-in software Bell Curve (Social Survey Research Information Co, Ltd) for Excel version 3.21(Microsoft Corporation) were used as tools for conducting statistical analyses.

The measurement time was from 9 AM to 4 PM, and the HR information and body vibration data of the operators were collected at any time. We collected 42 data sets from 9 participants measured in approximately 5 minutes, excluding preparation time and breaks. The measurement data of the operators in the working environment are presented in Multimedia Appendix 2. All participants were male and were asked to provide information on their age, height, and weight. As the cardiopulmonary function was intended to exclude unhealthy participants from the measurement, participants were also asked about their history of cardiovascular disease and their current health status.

We checked for the Hawthorne effect when participants were examined [22]. In this experiment, our research team did not monitor the participants' activities. It stayed away from the remote-control seat area and recorded the work using two cameras installed in the control area. Our study focused on the mental load that occurs during daily construction machine operation tasks. Therefore, before starting the measurements, we explained to the participants that this study was intended to measure the load during operation but not their operating skills, and we instructed them not to deviate from their daily operating mindset.

### HRV Metrics

HRV is associated with other aspects of health that are directly affected by autonomic function, such as self-regulation, and psychological and physiological stress [5,9,10]. A low HRV indicates inappropriate coordination between the sympathetic and parasympathetic nervous systems and is a reliable predictor of future cardiovascular disease [4,5]. Therefore, HRV measurements provide important information for assessing physical functioning and help identify the risk of physical fatigue and debilitation [23,24].

HR and HRV metrics have recently shown promise in multiple applications for health care providers [25-27]. Although studies performing HRV analysis are being reported since a long time, further improvements in technology and the interest of many researchers and physicians have brought more attention to this field [28]. Despite concerns about the validity of certain metrics of HRV data for measuring sympathetic balance [29,30] (eg, low frequency [LF] power of 0.04-0.15 Hz and the ratio of LF to high frequency [HF] power of 0.15-0.4 Hz in HRV), a number of previous studies support the notion that HRV analysis could reveal the balance of sympathetic and parasympathetic tones in the body [31]. The autonomic response to psychological stress has been studied using HRV [7]. Notable studies on HRV have emphasized the value of objective measures of stress in health care workers, and Joseph et al [27] found that self-reported stress was associated with proportionally elevated physiological levels of stress. These results provide compelling evidence for physicians, especially those who routinely perform surgical medical duties under tight time constraints, to assess their own stress. The widespread use of objective and ecologically valid measures of stress might provide important clues for understanding and reducing the psychological burden of stressful situations [32].

There are several widely accepted HRV metrics [32,33]. HRV measurements are classified into two categories: time-domain measures and frequency-domain measures. HRV metrics include the root mean square of the continuous difference (RMSSD; time domain), SD of the RRI (SDRR; time domain) and the LF/HF ratio (frequency domain). In a previous study involving corporate employees, RMSSD values were found to be related to perceived mental stress [34], with lower values indicating higher stress. The RMSSD metric is less sensitive to the number of missing data points. Therefore, the RMSSD can be seen as a more robust metric for evaluating patients with low data quality. The SDRR is calculated from the SD of normal RR intervals, and the lower the SDRR, the lower the HRV [33].

It is important to note that HRV measurements are derived from RR data and affected by the duration of the time series (number of data points), time of day, body orientation, and activity being performed. Where possible, these factors are derived using 5-minute RRIs and provide values for each activity, although Troubat et al [35] found that even brief periods of mental stress are associated with lower mean HRV values.

### MSE Metrics

MSE is an analytical algorithm that has gained popularity in the last 20 years to evaluate the complexity of time series at various time scales [36]. The physiological systems involved in maintaining stable health and well-being are complex and are affected by multiple interactions within and between system components. The complexity of the time series data being analyzed is reflected in the temporal structure of the variability of the output signal [37,38]. Entropy has been recognized as an excellent indicator of system complexity by applying and calculating the dynamics related to the HR, brain waves, and body sway [39]. Low entropy is associated with frailty, fatigue, aging, and functional impairment, whereas high entropy is associated with a greater ability to adapt to a changing environment [40,41]. Entropy has been reported as a reliable marker of neurophysiological complexity and adaptability in autonomic and somatic nervous systems [38]. In this study, the numerical value of entropy confirmed that adaptive capacity reduced because of task fatigue. The entropy value is obtained by plotting the entropy value of each coarse-grained time series as a function of the scale. The cardiac entropy index shows the area under the corresponding MSE curve (area calculated using the trapezoidal formula), and this area is treated as the entropy value [37,38,41].

Since its conception, the MSE algorithm has been applied to several analyses with significant success [42,43]. However, concerns have been raised about the statistical unreliability of the sample entropy of the coarse-grained series as the time scale factor of the MSE increases [44]. In recent years, a number of improved algorithms have been presented to address this concern, and these can be applied with satisfactory accuracy in the analysis of relatively small time series data sets having 750 points or less [41,43]. To calculate the complexity index, the time scale in our study was chosen from 1 to 14 [38]. In the analysis of the HR and MSE data of the participants, m=2 (vector length of time series) and r=0.15% (the similarity criterion used to compare vectors) of the SD of the original time series were used to calculate the sample entropy [38,43,44]; moreover, the refined composite multiscale entropy (RCMSE) [41] was used as the calculation algorithm of the MSE.

### Physical Vibration

Vibration occurs when the body is exposed to internal or external forces. Physical factors such as noise, heat, vibration, and radiation are environmental stressors with many stimuli that are detrimental to health and can alter bodily functions [45]. Vibrations can be harmful to human health, depending on the intensity and duration of exposure. The ISO (International Standards Organization) 2631-1 developed in 1997 [46] provides guidance on the use of methods to assess human exposure to vibration. For this purpose, frequency weighting and magnification of each evaluation axis are applied, as human response to vibration and its effects depend on the frequency of the vibration, its direction, and the studied effect (health, comfort, task) [45-47].

The transmission of vibrations from external systems to the human body has a significant impact on comfort, performance, and health. As the actual operation of the construction machinery and the remote-control seat including virtual reality (VR) are dynamic systems, the related transmission depends on the frequency and direction of the input motion. The transmission rate of vibration also depends on the characteristics of the seat exposed to the vibrations. On-road and off-road vehicles are exposed to vibrations caused by uneven road and soil profiles, and by moving elements in the machine. This is also the case for technical vehicles and wheelchair systems. Vibrations in the frequency range below 10-12 Hz affect the entire human body, whereas vibrations above 12 Hz have only localized effects [48]. LF (4-6 Hz) cyclical movements, such as vehicle tires rolling over an uneven road, may cause the body to resonate. Exposure to vibration in a seated position can cause muscle fatigue, weaken soft tissues, and increase the strain on the operator's back and whole body [49]. Continued external forced body vibration might lead to unpleasant symptoms such as lassitude, discomfort, and in severe cases, vomiting [48,50]. The vibration indices used for measuring physical vibration from the output of the three-axis accelerometer attached to the sensing wear while the machine operator sat on the remote-control seat are listed in Multimedia Appendix 3.

The physical vibration of the operator at the observation time (exposure time) T can be expressed by the root mean square weighted acceleration (Aw). The total vibration acceleration at each sample time, Aw(t), is the instantaneous value of the frequency-corrected acceleration (m/s^2^), and it is the composite of the accelerations along each axis occurring in the vertical, horizontal, and lateral directions. Further, acc is the vibration acceleration along each axis (m/s^2^) and a function of time. Aw is the basis for evaluating the effect of vibration on the human body according to ISO 2631. Health hazards and discomfort caused by vibration acceleration are affected not only by steady vibration but also by occasional shocks [45,46]. However, because Aw is an effective value and is averaged over the observation time, the impact of shocks can be possibly underestimated.

The vibration doses value (VDV) index defines the amount of vibration exposure. Instead of determining the change in acceleration over time by squaring the acceleration, it is determined by quadrature; the VDV determined using the root of the fourth power is more sensitive to peak values than the root of the second power; it is not averaged over the observation time and represents the entire vibration exposure during the observation time T [45,46].

The motion sickness dose value (MSDV_z)_ is calculated by correcting the vibration acceleration of the vertical axis of the operator using the frequency correction factor Wf [46,50]. MSDV_z_ is an index of vertical vibration of approximately 0.5-5 Hz, and experiments have shown that it is affected by the discomfort and stress of the ride in the passenger seat of the vehicle [48].

The vibration index of the operators' body vibration in this study is expressed using Aw, VDV, and MSDV_z_.

### Workload (Percentage Heart Rate Reserve [%HRR])

Hwang et al [51] suggested that caution should be exercised while sustaining a 30-40% HRR among construction workers, and Norton et al [52] suggested that a 40-60% HRR lasting 30-60 minutes is equivalent to a moderate physical load for adequate health care of sedentary persons. Compared to construction workers (eg, scaffolders and steel handlers), who are often exposed to physical loads that exceed workload limits, construction equipment operators are exposed to higher psychological loads and stresses. Although psychological load has a negligible effect on the HR when measured over a long period, it may affect the %HRR for a short period of time [52].

HRR is a measure of the workload or pressure intensity at work, associated with muscle activity [53]. Equation 1 depicts how it is estimated:

HRR = (HR_working_ – HR_resting_) / (HR_maximum_ – HR_resting_) × 100 (%) **(1)**

where HR_working_ is the mean working heart rate, HR_resting_ it the resting heart rate, and HR_maximum_ is the maximum heart rate based on age [51,53].

### Removal of Artifacts

Two types of outliers are commonly found in heartbeat interval time series because of error beats and artifacts. These outliers have no physiological significance. However, artifacts can significantly distort measurements in the time and frequency domains, increasing the power in all frequency bands [37]. For HRV data, a value can be considered valid if the clean segment is long enough in the time series to calculate the power in the frequency band. For example, it has been pointed out that at least 2.5 minutes of clean data is needed to estimate LF power [54]. Furthermore, for MSE, if the RRIs of the heartbeats differ by several orders of magnitude from the mean of the time series, it may have a significant impact on the entropy calculation [37]. The data set collected in this research was filtered to exclude artifacts, ventricular extrasystoles, and undetected heartbeats [37,44,55]. Briefly, at the center point of a moving window of length l, anything outside the interval 
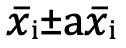
 was excluded. 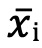 represents the mean of the data points within that moving window, calculated excluding the center point, and a is a positive number less than or equal to 1. In this study, we used l=41 and a=0.2 [37,44,55].

### Hypotheses Development

Based on the research objectives and literature review, the following hypotheses were developed:

H1: In the driving time of the construction machine, differences in the operating environments during the riding operation, remote operation using the monitor image, and remote operation by VR appear in the parameters of the HRV and MSE.

H2: The magnitude of vibration in the operating environment has a negative relationship with the parameters of the HRV and MSE.

H3: The driving time of the construction machine has a negative relationship with the magnitude of vibration of the operating environment and the parameters of the HRV and MSE.

## Results

### Normality Tests for Data

The normality of the collected data was evaluated. When the sample size is greater than 50, only the Kolmogorov-Smirnov test is suitable to determine normality [56]. However, in this research, the sample size was not sufficiently large, and the data for which the normality could be confirmed using the Shapiro-Wilk and Kolmogorov-Smirnov tests were considered to be normally distributed (see Multimedia Appendix 4). In both the tests, the null hypothesis assumes that the dataset is normally distributed, with an alpha null hypothesis going further to assume that the data set is normally distributed with *P*=.05 [57]. The data on LF, MSE, %HRR, and VDV satisfied the conditions of normal distribution.

### Descriptive Statistics and Intergroup Comparisons

Descriptive statistics, means, and SDs were used to determine if there were any significant differences among the data collected for each group in the three operating environments: actual machine operation, remote operation using only monitor images, and VR operating environment. For the analysis of normally distributed data, multiple comparisons using the Bonferroni test were employed in the primary allocation analysis of variance. For the analysis of non-normally distributed data, multiple comparisons using the Steel-Dwass method in the Kruskal-Wallis test were conducted. The results of the analysis are presented in Table 1.

The Kruskal-Wallis test does not require a normal distributed dataset [58]. Its null hypothesis is that there is “no difference between the three groups” at a significance level of .05. If *P*<.05, the null hypothesis is rejected, indicating that there is a statistically significant difference in the means of the different groups. The Bonferroni test has the same hypothesis as the Kruskal-Wallis test, but it relies on the assumptions of normality and homogeneity of the population [59].

The HRV time-domain parameter of HR RRI, and HRV frequency-domain parameters of LF, LF/HF, HR variability, complexity of MSE, workload %HRR, body vibration Aw, VDV, MSDV_z_, and driving time of the construction equipment were statistically significant between the two operating environments. Statistically significant differences were also found between the three operating environments for the LF/HF HRV parameters in the frequency domain, Aw of physical vibration, MSDV_z_, and driving time of the construction equipment.

The riding operation of the construction machine resulted in the highest stress indices, LF/HF HRV, physical vibration Aw, and MSDV_z_, and the shortest driving time. The riding operation shortened the driving time, but it increased the operator's stress. In contrast, remote control using the monitor image showed the smallest LF/HF, Aw, and MSDV_z_, and the longest driving time. Thus, in remote operation using monitor images, the stress of the operator was lower, but the driving time was longer.

**Table 1:** Mean, variance, and *P* value of each parameter for heart rate variability, physical workload, work vibration, and machine operation.

Parameter		Riding operation	Remote operation	VR^a^ operation	Norm test	*P* value between each operation		
						1-2	2-3	1-3
**HRV^b^ time domain, mean (SD)**								
	RRI^c^ (ms^d^)	664.8 (92.6)	828.9 (140)	803.0 (152)	NP^e^	.008	.05	.85
	SDRR^f^ (ms)	66.5 (22.6)	70.7 (36.3)	60.0 (22.0)	NP	.96	.66	.72
	RMSSD^g^ (ms)	24.2 (7.96)	25.3 (10.1)	26.4 (8.02)	NP	.90	.57	.78
**HRV frequency domain, mean (SD)**								
	LF^h^_nu_	77.2 (9.44)	63.6 (9.70)	73.0 (13.0)	P^i^	<.001	.005	.38
	LF _power_	31.1 (7.13)	29.9 (8.69)	32.7 (5.36)	NP	.87	.63	.98
	LF/HF^j^	4.72 (1.52)	1.85 (0.78)	3.27 (0.99)	NP	<.001	.004	.04
	MSE^k^	7.13 (1.26)	11.3 (2.78)	6.88 (2.10)	P	<.001	<.001	.99
**Physical workload, mean (SD)**								
	%HRR^l^	13.0 (6.30)	3.70 (4.01)	5.17 (5.45)	P	<.001	.001	.99
**Work vibration, mean (SD)**								
	Aw^m^	60.7 (18.1)	11.6 (12.8)	56.5 (21.4)	NP	<.001	<.001	.004
	VDV^n^	5678 (4067)	1.80 (2.25)	828.2 (2377)	P	<.001	.99	<.001
	MSDV_z_^o^	133.9 (2.59)	126.5 (2.26)	131.3 (2.56)	NP	<.001	<.001	.04
**Machine operation, mean (SD)**								
	Running time	306.3 (48.7)	436.2 (81.7)	369.8 (77.6)	NP	<.001	.04	.05

^a^VR: virtual reality.

^b^HRV: heart rate variability.

^c^RRI: RR interval.

^d^ms: milliseconds.

^e^NP: nonparametric.

^f^SDRR: SD of RRI.

^g^RMSSD: root mean square of the continuous difference.

^h^LF: low frequency.

^i^P: parametric.

^j^HF: high frequency.

^k^MSE: multiscale entropy.

^l^HRR: heart rate reserve.

^m^Aw: vibration index.

^n^VDV: vibration doses value.

^o^MSDV_z_: motion sickness dose value.

### Relationships Between Psychological and Working Loads, and Physical Vibration

Data collected in the three operating environments were combined to analyze their effects on the psychological load and workload. Multiple regression analysis was conducted to evaluate the significant relationships between the psychological load, workload, and physical vibration. The results are presented in Table 2. In the multiple regression analysis, we checked for multicollinearity in the independent variables (indicators of physical vibration). All three indices of physical vibration, (Aw, VDV, and MSDV_z_) had a variance inflation factor (VIF) less than 10. Subsequently, a significant relationship was found between the operational load and body vibration.

As shown in Table 3, the large oscillation of the VDV determined by the quadrature oscillation dose method has a positive effect on the workload %HRR. Additionally, the time average of the root mean square weighted acceleration, Aw, had a positive effect on the stress index, LF/HF HRV, as observed in Table 4.

Aw had a negative effect on the MSE, a measure of adaptability inferred from the complexity of the heartbeats, as indicated in Table 5. The adjusted R^2^ value for this regression equation was 0.189.

These results show that the %HRR has a significant relationship with the VDV. However, the other vibration indices, Aw and MSDV_z_, did not show any significant relationship with the %HRR. LF/HF and MSE showed significant relationships with Aw. The effects of some vibration indices on the %HRR, which indicates the workload in the operating environment, LF/HF, which indicate the psychological load, and MSE, are shown.

**Table 2:** Relationship between the independent variable (work vibration) and dependent variables (workload, low frequency/high frequency, and multiscale entropy).

Independent variables			Dependent variables																	
			%HRR^a^						LF^b^/HF^c^							MSE^d^				
			β^e^		SE		*P* value		β		SE		*P* value		β			SE		*P* value
**Work vibration**																				
	Aw^f^	–0.002		0.039		.96		0.0238		0.0093		.01		–0.031			0.0175		.37	
	VDV^g^	0.0006		0.0003		.06		0.000		0.0001		.82		–0.0001			0.0001		.24	
	MSDV_z_^h^	0.515		0.294		.09		0.0983		0.0693		.16		–0.156			0.130		.08	

^a^HRR: heart rate reserve.

^b^LF: low frequency.

^c^HF: high frequency.

^d^MSE: multiscale entropy.

^e^β: beta coefficient.

^f^Aw: vibration indices.

^g^VDV: vibration doses value.

^h^MSDV_z_: motion sickness dose value.

**Table 3:** Relationships between independent variable (work vibration) and dependent variable (workload).

Model 1-1: independent variable	Dependent variable: %HRR^a^			
	Estimated	SE	*t* value^b^	*P* value
VDV^c^	0.0008	0.0003	3.29	.002
Intercept	5.469	1.07	5.11	<.001
Multiple R^2d^	0.213	—^e^	—	—
Adjusted R^2^	0.193	—	—	—
*F* static value^f^	10.8	—	—	.002

^a^HRR: heart rate reserve.

^b^*t* value: result of the student *t* test.

^c^VDV: vibration doses value.

^d^R^2^: coefficient of determination.

^e^Not available

^f^*F* static value: variance ratio.

**Table 4:** Relationship between independent variables (work vibration) and dependent variables (low frequency/high frequency).

Model 1-2: independent variable	Dependent variable: LF^a^/HF^b^			
	Estimated	SE	*t* value^c^	*P* value
Aw^d^	0.0329	0.0079	4.18	<.001
(Intercept)	1.817	0.405	4.49	<.001
Multiple R^2e^	0.305	—^f^	—	—
Adjusted R^2^	0.287	—	—	—
*F* static value^g^	17.5	—	—	<.001

^a^LF: low frequency.

^b^HF: high frequency.

^c^*t* value: result of the student's *t* test.

^d^Aw: vibration indices.

^e^R^2^: coefficient of determination.

^f^Not available

^g^*F* static value: variance ratio.

**Table 5:** Relationship between independent variables (work vibration) and dependent variables (multiscale entropy).

Model 1-3: independent variable	Dependent variable: MSE^a^			
	Estimated	SE	*t* value^b^	*P* value
Aw^c^	–0.0485	0.00149	–3.24	.02
Intercept	10.55	0.768	13.7	<.001
Multiple R^2d^	0.208	—^e^	—	—
Adjusted R^2^	0.189	—	—	—
*F* static value^f^	10.5	—	—	.002

^a^MSE: multiscale entropy.

^b^*t* value: result of the student *t* test.

^c^Aw: vibration indices.

^d^R^2^: coefficient of determination.

^e^Not available.

^f^*F* static value: variance ratio.

### Significant Statistic of Each Parameter for Driving Time

Data collected in the three operating environments were combined, and multiple regression analysis was performed to evaluate the relationships among several HRV indices and the parameters of MSE, HR RRI, and physical vibration, which indicated the complexity of HR changes, with the driving time of construction equipment. Two multiple regression equations were used to confirm a statistically significant relationship. First, in the multiple regression analysis, we found no multicollinearity among the independent variables. As a result, it was confirmed that the parameters among the two sets of dependent variables used, namely, LF/HF, RRI, MSDV_z_, and MSE, and RRI and MSDV_z_, had VIFs between 1 and 2, and there was no possibility of multicollinearity. In the subsequent analysis of the physical and psychological loads and physical vibrations during the operation, two significant relationships were found.

The first was the effect of the explanatory variables LF/HF, HR RRI, and oscillation MSDV_z_ on the driving time; the adjusted R^2^ of this regression equation was 0.449. Second, the driving time was affected by the explanatory variables, namely MSE (complexity of HR change), HR RRI, and vibration MSDV_z_; the adjusted R^2^ of this regression equation was 0.400. The results of the analysis are presented below in Tables 6 and 7.

Multiple regression analysis of the data for each construction machine operation suggested that the driving time affects LF/HF, which indicates operational stress, and MSE, which indicates adaptability; it also affects the RRI and vibration index MSDV_z_. Equations 2 and 3 are the multiple regression equations obtained for the driving time HRV and driving time MSE. In both these regression equations, as the construction machine runs faster and the driving time becomes shorter, the operator's stress increases, the adaptability to the task decreases, and the RRI and MSDV_z_ also increase.

Driving time_HRV_ = –24.5 × LF/HF – 0.350 × RRI – 10.7 × MSDV_z_ + 2115 **(2)**

Driving time_MSE_ = 10.5 × MSE – 0.259 × RRI – 10.3 × MSDV_z_ + 1823 **(3)**

**Table 6:** Relationship among independent variables (low frequency/high frequency, RR interval, and work vibration) and dependent variable (driving time).

Independent variables	Dependent variable: driving time				
	Estimated	SE	*t* value^a^	*P* value	VIF^b^
LF^c^/HF^d^	–24.5	7.32	–3.34	.002	1.40
RRI^e^	–0.350	0.0814	–4.30	<.001	1.40
MSDV_z_^f^	–10.7	3.07	–3.49	.001	1.44
(Intercept)	2115.6	420.0	5.04	<.001	—^g^
Multiple R^2h^	0.490	—	—	—	—
Adjusted R^2^	0.449	—	—	—	—
*F* static value^i^	12.1	—	—	<.001	—

^a^*t* value: result of the student *t* test.

^b^VIF: variance inflation factor.

^c^LF: low frequency.

^d^HF: high frequency.

^e^RRI: RR interval.

^f^MSDV_z_: motion sickness dose value.

^g^Not available.

^h^R^2^: coefficient of determination.

^i^*F* static value: variance ratio.

**Table 7:** Relationship among independent variables (multiscale entropy, RR interval, and work vibration) and dependent variable (driving time).

Independent variables	Dependent variable: driving time				
	Estimated	SE	*t* value^a^	*P* value	VIF^b^
MSE^c^	10.5	4.01	2.63	.01	1.27
RRI^d^	–0.259	0.0807	–3.21	.003	1.24
MSDV_z_^e^	–10.3	3.31	–3.11	.004	1.53
Intercept	1823.3	476.6	3.83	<.001	—^f^
Multiple R^2g^	0.444	—	—	—	—
Adjusted R^2^	0.400	—	—	—	—
*F* static value^h^	10.1	—	—	<.001	—

^a^*t* value: result of the student *t* test.

^b^VIF: variance inflation factor.

^c^MSE: multiscale entropy.

^d^RRI: RR interval.

^e^MSDV_z_: motion sickness dose value.

^f^Not available.

^g^R^2^: coefficient of determination.

^h^*F* static value: variance ratio.

### Driving Construction Machines With Acceptable Operational Stress

Using equation 2 regarding the driving time of construction machinery obtained in this study, we suggest reducing the driving time of the construction machine while suppressing the operation stress. Equation 2 shows the relationship between the physical vibration and psychological load during machine operation, and it is expected that reducing the physical vibration during operation will reduce the operational stress. By reducing the stress caused by Aw, the time average of the squared vibration, to an acceptable level, it is possible to reduce the driving time and operator stress.

**Figure 2:**
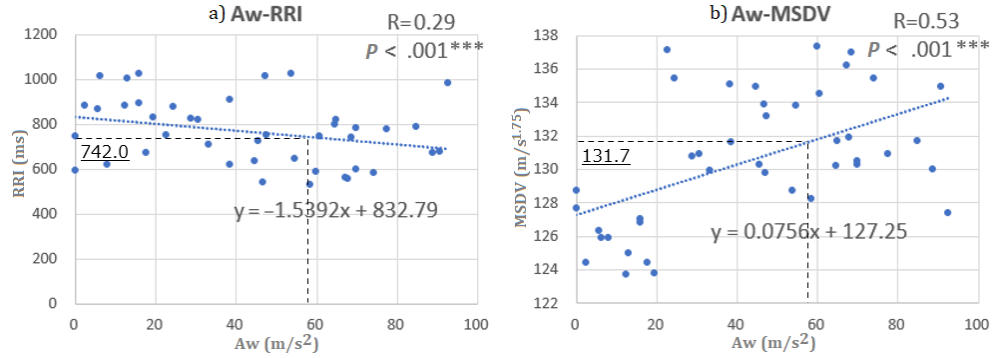
A. Correlation between RR interval and vibration index Aw and B. Correlation between motion sickness doses value and vibration index Aw. The dashed lines show the estimated values of the RR interval and motion sickness dose value for the allowed Aw. In the figure, the linear relationship equation, correlation coefficient R, and *P* values related to the two axes are shown. MSDV: motion sickness dose value; RRI: RR interval.

There are no previous reports where LF/HF for stress levels has been quantitatively determined. In this study, an LF/HF of value 2 was considered an acceptable stress level based on reports investigating the stress of participants in a sitting posture [60-62]. Using the relationship presented in Table 5, the average squared vibration acceleration during traveling Aw_LF/HF=2_ is approximately 59 m/s^2^ according to Aw = 2/0.0329 – 1.82. From the relationship between equation 2 and Figure 2, the traveling time of the construction machine = –24.5 × 2 – 0.35 × 742.0 –10.7 × 131.7 + 2115 = 397.1 seconds (approximately), which is the driving time for one lap that is acceptable for the psychological load of the operator. It was also estimated that the running speed at an acceptable psychological load = 400 m /397.1 s ≈ 1.01 m/s ≈ 3.63 km/h.

## Discussion

### Principal Findings

In this research, we investigated the effects of stress on psychological health during the operation of construction equipment, and the relationship between the appropriate stress and the driving time of construction machines. We found a significant relationship between LF/HF HRV [7,31] and MSE [6,40-42], which indicates the complexity of the HR, and body vibration [48-50]; thus, our hypothesis was supported. In addition, it was found that by keeping the operational stress caused by Aw, which is the time average of the squared vibration acceleration, at an acceptable level, an appropriate driving time that takes the operator into account can be obtained.

### Stress and Remote Operation

Indices related to stress characterize the activity of the sympathetic part of the autonomous nervous system and can be appropriately applied to estimate psychological load as well as the intensity of physical workload [63]. Therefore, a similar relationship can be inferred between several HRVs related to stress and MSE. This is evident in the relationships shown in equations 2 and 3. The study results show that the uncertainty of what constitutes an acceptable limit of psychological load can be resolved by analyzing the stress index and some vibration indices in the working environment. The operating technicians were exposed to different stress levels owing to the physical vibrations of the construction equipment. In addition, the workload by %HRR was found to be related to physical vibration, but not to the driving time. Riding operators with the highest vibration exhibited multiple lower HRV indices and MSEs than operators exposed to stress when remotely controlling the construction machine with only monitor images, which had the lowest vibration. Therefore, the hypothesis that the physical vibration experienced by construction workers has a negative effect on the HRV and MSE was supported. In addition, the hypothesis that the driving time of the construction machine has a negative relationship with the magnitude of vibration in the operating environment, and the parameters of the HRV and MSE, was also confirmed.

In an environment with large vibrations, the operator has lower multiple HRV indices because of a higher sympathetic nervous system tone (LF power) and sympathetic balance (LF/HF), and a lower parasympathetic nervous system tone (ie, RRI, SDRR, RMSSD, and HF power) [33]. This result is consistent with previous studies estimating that increased work stress is associated with lower parasympathetic activation as sympathetic activity increases [64,65]. LF/HF provides insight into the stress classification of participants. Operators in this study were exposed to a low physical load and high mental stress. This is in comparison to physical workers who engage in production through physical activity, which may result in higher psychological load due to the nature of their work, as it involves paying full attention to the safety of their surroundings and ensuring work quality through machine operation. The results of this study reflect the findings of Boschman et al [66]. Operators experience high psychological job demands and a high need for recovery. Hence, job-specific psychosocial work factors need to be assessed.

The measurement system and sensing wear used in this study are reliable [67] and provide valid HRV data. However, care should be taken when using them for implementing frequency-domain analysis to interpret cardiac autonomic modulation [68]. For accurate measurement of indices related to LFs and HFs in the frequency domain, continuous recording with a stable HR measurement period of at least 3 minutes is recommended [5]. This application can be socially implemented as a useful tool for monitoring the cardiac autonomic health status of operation workers. It is useful in managing the stress levels of operation technicians during machine operations by efficiently using short-term HRV and MSE beat information and body vibration recordings.

### Theoretical and Practical Contributions

This study contributes theoretically by demonstrating the influence of psychological load as measured by the HRV and MSE on the operation of construction machines, and the effect of the psychological load of skilled workers on the HR interval, vibration in the working environment, and driving time of the construction machine. In addition, the study presents a new relational model using biometric information on HR and vibration indices in the work environment for the driving time of construction equipment.

Regarding the practical contributions, we quantified the vibration in the work environment of the driving operation and clarified the psychological workload of the operation. The evidence connecting the physical vibration in the work environment and the psychological and physical fatigue of workers could cause construction companies to improve their working environment and workforce management [69]. Furthermore, a new concept considering the psychology of the operator and the efficiency of the operation from the perspective of health psychology was introduced by comparing riding operation and remote operations, assuming a construction site where construction machines could not be operated.

### Limitations

This study had several limitations. First, the number of construction machine operators employed was disproportionate; hence, the age and gender of the operators were not considered. There are reports that stress varies with age and gender [70,71], but this study was conducted on healthy males aged 18-50 years; hence, the study results may not be generalizable to all technicians in the construction industry. The number of male workers in the Japanese construction industry is very high, and further research might be beneficial in countries where there are a promising number of women in the construction and operations engineering professions. Second, this study was a cross-sectional analysis, and data were collected from the operators over 3 days. The collection of data over a longer period may provide more definitive results. Third, it would be desirable to analyze the operability and productivity of construction machines in relation to stress, as this research was limited to evaluation considering the driving time. The present study was conducted on construction machines that are typically used in construction work. A study of the psychological load during operation using machines with more fine-grained operational needs and controls could provide a comparison of the effects on the operator. Finally, as frequency-based metrics have been reported to represent the balance between sympathetic and parasympathetic activities more accurately [72], it is critical to improve the quality of HR interval recordings in wearable devices. Among the data collected in this research, there were some missing heartbeat intervals, which affected the selection of HRV metrics and necessitated the removal of a sample of participants from statistical analysis. To conduct a large sample study over a long period, future research aimed at furthering sensing wear and wearable technologies such as the WHS-2 to improve recording quality (eg, further minimizing motion artifacts) is essential. This will enhance the usefulness of the devices used.

### Conclusions

A new method was developed in this study to calculate the appropriate operating time considering operational stress and maintaining the physical vibration within an acceptable range. The participants had to be alert while operating the machine in an environment that could expose them to high stress from vibration. Although this research is based on a limited number of participants in a special environment, by focusing on the relationship between psychological load and physical vibration, which remains unexplored in previous studies, the relationship of these variables with the operation time of construction machines was clarified.

## Appendix

Multimedia Appendix 1 List of devices and infrastructure used for the measurements of heart rate and acceleration.

Multimedia Appendix 2 Measurement data of the operators collected in the work environment.

Multimedia Appendix 3 Measurement indices for physical vibrations (root mean square weighted acceleration, vibration doses value , and motion sickness dose value) in operating work.

Multimedia Appendix 4 Results of normality tests for heart rate variability and vibration specifications (root mean square weighted acceleration, vibration dose value, and motion sickness dose value).

## Abbreviations

ECG: electrocardiogram
HF: high frequency
HR: heart rate
HRR: heart rate reserve
HRV: heart rate variability
ISO: International Standards Organization
LF: low frequency
MSDV: motion sickness dose value
MSE: multiscale entropy
RMSSD: root mean square of the continuous difference
RRI: RR interval
SDRR: standard deviation of the RR intervals
VDV: vibration doses value
VIF: variance inflation factor
VR: virtual reality

## References

[1] Chikushi S, Moriyama Y, Fujii H, Tamura Y, Yamakawa H, Nagatani K, Sakai Y, Chiba T, Yamamoto S, Chayama K, Yamashita A, Asama H (2020). Automated image presentation for backhoe embankment construction in unmanned construction site. Proceedings of the 2020 IEEE/SICE International Symposium on System Integration.

[2] Tripicchio P, Ruffaldi E, Gasparello P, Eguchi S, Kusuno J, Kitano K, Yamada M, Argiolas A, Niccolini M, Ragaglia M, Avizzano Ca (2017). A Stereo-Panoramic Telepresence System for Construction Machines. Procedia Manufacturing.

[3] Brandt M, Madeleine P, Samani A, Ajslev JZ, Jakobsen MD, Sundstrup E, Andersen LL (2018). Effects of a participatory ergonomics intervention with wearable technical measurements of physical workload in the construction industry: cluster randomized controlled trial. J Med Internet Res.

[4] Ernst G (2017). Heart-rate variability-more than heart beats?. Front Public Health.

[5] Shaffer F, Ginsberg JP (2017). An overview of heart rate variability metrics and norms. Front Public Health.

[6] Tiwari A, Albuquerque I, Parent M, Gagnon J, Lafond D, Tremblay S, Falk TH (2019). Multi-scale heart beat entropy measures for mental workload assessment of ambulant users. Entropy (Basel).

[7] Castaldo R, Melillo P, Bracale U, Caserta M, Triassi M, Pecchia L (2015). Acute mental stress assessment via short term HRV analysis in healthy adults: a systematic review with meta-analysis. Biomed Signal Process Control.

[8] Wen W, Liu G, Mao Z, Huang W, Zhang X, Hu H, Yang J, Jia W (2020). Toward constructing a real-time social anxiety evaluation system: exploring effective heart rate features. IEEE Trans Affective Comput.

[9] Patel M, Lal S, Kavanagh D, Rossiter P (2011). Applying neural network analysis on heart rate variability data to assess driver fatigue. Expert Syst Appl.

[10] Salahuddin L, Cho J, Jeong M, Kim D (2007). Ultra short term analysis of heart rate variability for monitoring mental stress in mobile settings. Proceedings of the 9th Annual International Conference of the IEEE Engineering in Medicine and Biology Society.

[11] Landreani F, Martin-Yebra A, Casellato C, Frigo C, Pavan E, Migeotte P, Caiani EG (2016). Beat-to-beat heart rate detection by smartphone's accelerometers: validation with ECG. Proceedings of the 38th Annual International Conference of the IEEE Engineering in Medicine and Biology Society (EMBC).

[12] Hashiguchi N, Kodama K, Lim Y, Che C, Kuroishi S, Miyazaki Y, Kobayashi T, Kitahara S, Tateyama K (2020). Practical judgment of workload based on physical activity, work conditions, and worker's age in construction site. Sensors (Basel).

[13] Castaldo R, Montesinos L, Melillo P, James C, Pecchia L (2019). Ultra-short term HRV features as surrogates of short term HRV: a case study on mental stress detection in real life. BMC Med Inform Decis Mak.

[14] Melo HM, Martins TC, Nascimento LM, Hoeller AA, Walz R, Takase E (2018). Ultra‐short heart rate variability recording reliability: the effect of controlled paced breathing. Ann Noninvasive Electrocardiol.

[15] Matsuura H, Mukaino M, Otaka Y, Kagaya H, Aoshima Y, Suzuki T, Inukai A, Hattori E, Ogasawara T, Saitoh E (2019). Validity of simplified, calibration-less exercise intensity measurement using resting heart rate during sleep: a method-comparison study with respiratory gas analysis. BMC Sports Sci Med Rehabil.

[16] Holmes TH, Rahe RH (1967). The social readjustment rating scale. J Psychosom Res.

[17] Lazarus R, Cohen J (1997). Environmental stress. Human Behavior and Environment.

[18] Chelidoni O, Plans D, Ponzo S, Morelli D, Cropley M (2020). Exploring the effects of a brief biofeedback breathing session delivered through the biobase app in facilitating employee stress recovery: randomized experimental study. JMIR Mhealth Uhealth.

[19] Selye H (1936). A syndrome produced by diverse nocuous agents. Nature.

[20] Selye H (1978). Stress of Life 2nd edition.

[21] Ministry of Education, Culture, Sports, Science and Technology—Japan.

[22] Adair JG (1984). The Hawthorne effect: a reconsideration of the methodological artifact. J Appl Psychol.

[23] Mccraty R, Shaffer F (2015). Heart rate variability: new perspectives on physiological mechanisms, assessment of self-regulatory capacity, and health risk. Glob Adv Health Med.

[24] Graham SA, Jeste DV, Lee EE, Wu T, Tu X, Kim H, Depp CA (2019). Associations between heart rate variability measured with a wrist-worn sensor and older adults' physical function: observational study. JMIR Mhealth Uhealth.

[25] Amirian I, Toftegård Andersen L, Rosenberg J, Gögenur I (2014). Decreased heart rate variability in surgeons during night shifts. Can J Surg.

[26] Rieger A, Stoll R, Kreuzfeld S, Behrens K, Weippert M (2014). Heart rate and heart rate variability as indirect markers of surgeons' intraoperative stress. Int Arch Occup Environ Health.

[27] Joseph B, Parvaneh S, Swartz T, Haider AA, Hassan A, Kulvatunyou N, Tang A, Latifi R, Najafi B, Rhee P (2016). Stress among surgical attending physicians and trainees: a quantitative assessment during trauma activation and emergency surgeries. J Trauma Acute Care Surg.

[28] Billman GE (2011). Heart rate variability - a historical perspective. Front Physiol.

[29] Reyes del Paso GA, Langewitz W, Mulder LJM, van Roon A, Duschek S (2013). The utility of low frequency heart rate variability as an index of sympathetic cardiac tone: a review with emphasis on a reanalysis of previous studies. Psychophysiology.

[30] Billman GE (2013). The LF/HF ratio does not accurately measure cardiac sympatho-vagal balance. Front Physiol.

[31] Malliani A, Pagani M, Lombardi F, Cerutti S (1991). Cardiovascular neural regulation explored in the frequency domain. Circulation.

[32] Peters GA, Wong ML, Joseph JW, Sanchez LD (2019). Pulse rate variability in emergency physicians during shifts: pilot cross-sectional study. JMIR Mhealth Uhealth.

[33] Nwaogu JM, Chan AP (2021). Work-related stress, psychophysiological strain, and recovery among on-site construction personnel. Autom Constr.

[34] Orsila R, Virtanen M, Luukkaala T, Tarvainen M, Karjalainen P, Viik J, Savinainen M, Nygård C (2008). Perceived mental stress and reactions in heart rate variability--a pilot study among employees of an electronics company. Int J Occup Saf Ergon.

[35] Troubat N, Fargeas-Gluck M, Tulppo M, Dugué B (2009). The stress of chess players as a model to study the effects of psychological stimuli on physiological responses: an example of substrate oxidation and heart rate variability in man. Eur J Appl Physiol.

[36] Costa M, Goldberger AL, Peng C (2002). Multiscale entropy analysis of complex physiologic time series. Phys Rev Lett.

[37] Costa M, Goldberger AL, Peng C (2005). Multiscale entropy analysis of biological signals. Phys Rev E Stat Nonlin Soft Matter Phys.

[38] Blons E, Arsac L, Gilfriche P, Deschodt-Arsac V (2019). Multiscale entropy of cardiac and postural control reflects a flexible adaptation to a cognitive task. Entropy.

[39] Busa MA, van Emmerik RE (2016). Multiscale entropy: a tool for understanding the complexity of postural control. J Sport Health Sci.

[40] Manor Brad, Costa Madalena D, Hu Kun, Newton Elizabeth, Starobinets Olga, Kang Hyun Gu, Peng C K, Novak Vera, Lipsitz Lewis A (2010). Physiological complexity and system adaptability: evidence from postural control dynamics of older adults. J Appl Physiol (1985).

[41] Wu S, Wu C, Lin S, Lee K, Peng C (2014). Analysis of complex time series using refined composite multiscale entropy. Phys Lett A.

[42] Pan J, Hu H, Liu X, Hu Y (2015). Multiscale entropy analysis on human operating behavior. Entropy.

[43] Wu S, Wu C, Lin S, Wang C, Lee K (2013). Time series analysis using composite multiscale entropy. Entropy.

[44] Costa M, Goldberger A (2015). Generalized multiscale entropy analysis: application to quantifying the complex volatility of human heartbeat time series. Entropy.

[45] Duarte MLM, de Araújo PA, Horta FC, Vecchio SD, de Carvalho LAP (2018). Correlation between weighted acceleration, vibration dose value and exposure time on whole body vibration comfort levels evaluation. Safety Science.

[46] (1997). ISO/WD 2631-1: Mechanical vibration and shock — Evaluation of human exposure to whole-body vibration — Part 1: General requirements. International Organization for Standardization.

[47] Subashi G, Nawayseh N, Matsumoto Y, Griffin M (2009). Nonlinear subjective and dynamic responses of seated subjects exposed to horizontal whole-body vibration. J Sound Vib.

[48] Hostens I, Papaioannou Y, Spaepen A, Ramon H (2003). A study of vibration characteristics on a luxury wheelchair and a new prototype wheelchair. J Sound Vib.

[49] Pope M, Magnusson M, Wilder D (1998). Lowback pain and whole-body vibration. Clin Orthop Relat Res.

[50] Els P (2005). The applicability of ride comfort standards to off-road vehicles. J Terramech.

[51] Hwang S, Lee S (2017). Wristband-type wearable health devices to measure construction workers' physical demands. Autom Constr.

[52] Norton K, Norton L, Sadgrove D (2010). Position statement on physical activity and exercise intensity terminology. J Sci Med Sport.

[53] Ismaila SO, Oriolowo KT, Akanbi OG (2013). Cardiovascular strain of sawmill workers in South-Western Nigeria. Int J Occup Saf Ergon.

[54] Kuusela T, Kamath MV, Watanabe MA, Upton ARM (2013). Methodological aspects of heart rate variability analysis. Heart Rate Variability (HRV) Signal Analysis.

[55] Behar JA, Rosenberg AA, Weiser-Bitoun I, Shemla O, Alexandrovich A, Konyukhov E, Yaniv Y (2018). PhysioZoo: a novel open access platform for heart rate variability analysis of mammalian electrocardiographic data. Front Physiol.

[56] Mishra P, Pandey C, Singh U, Gupta A, Sahu C, Keshri A (2019). Descriptive statistics and normality tests for statistical data. Ann Card Anaesth.

[57] Darko A, Chan APC (2018). Strategies to promote green building technologies adoption in developing countries: the case of Ghana. Build Environ.

[58] McKight P, Najab J (2010). Kruskal-Wallis test. The Corsini Encyclopedia of Psychology.

[59] Abdi H (2007). The Bonferonni and Šidák corrections for multiple comparisons. Encyclopedia of Measurement and Statistics.

[60] Skibniewski FW, Dziuda Ł, Baran PM, Krej MK, Guzowski S, Piotrowski MA, Truszczyński OE (2015). Preliminary results of the LF/HF ratio as an indicator for estimating difficulty level of flight tasks. Aerosp Med Hum Perform.

[61] Seong H, Lee J, Shin T, Kim W, Yoon Y, Yoon Y (2004). The analysis of mental stress using time-frequency distribution of heart rate variability signal. Proceedings of the 26th Annual International Conference of the IEEE Engineering in Medicine and Biology Society.

[62] Subahni A, Xia L, Malik A (2012). Association of mental stress with video games. Proceedings of the 4th International Conference on Intelligent and Advanced Systems.

[63] Quendler E, Trieb K, Nimmerichter A (2017). Validation of automated detection of physical and mental stress during work in a Hühnermobil 225. Ann Agric Environ Med.

[64] Garza JL, Cavallari JM, Eijckelhof BHW, Huysmans MA, Thamsuwan O, Johnson PW, van der Beek AJ, Dennerlein JT (2014). Office workers with high effort–reward imbalance and overcommitment have greater decreases in heart rate variability over a 2-h working period. Int Arch Occup Environ Health.

[65] Järvelin-Pasanen S, Sinikallio S, Tarvainen MP (2018). Heart rate variability and occupational stress-systematic review. Ind Health.

[66] Boschman J, van der Molen H, Sluiter J, Frings-Dresen M (2013). Psychosocial work environment and mental health among construction workers. Appl Ergon.

[67] Discover TOYOBO’s New Materials.

[68] Chen Y, Lu W, Pagaduan JC, Kuo C (2020). A novel smartphone app for the measurement of ultra-short-term and short-term heart rate variability: validity and reliability study. JMIR Mhealth Uhealth.

[69] Hashiguchi N, Cao J, Lim Y, Kubota Y, Kitahara S, Ishida S, Kodama K (2020). The effects of psychological factors on perceptions of productivity in construction sites in Japan by worker age. Int J Environ Res Public Health.

[70] Rudolph KD, Hammen C (1999). Age and gender as determinants of stress exposure, generation, and reactions in youngsters: a transactional perspective. Child Dev.

[71] Folkman S, Lazarus RS, Pimley S, Novacek J (1987). Age differences in stress and coping processes. Psychology and Aging.

[72] Singh N, Moneghetti KJ, Christle JW, Hadley D, Plews D, Froelicher V (2018). Heart rate variability: an old metric with new meaning in the era of using mhealth technologies for health and exercise training guidance. part one: physiology and methods. Arrhythm Electrophysiol Rev.

