# Supplementary material for: Psychological Effects of Heart Rate and Physical Vibration on the Operation of Construction Machines: Experimental Study
Source: JMIR Mhealth Uhealth. 2021 Sep 15;9(9):e31637. doi: 10.2196/31637 (PMC8482169; doi:10.2196/31637)
Supplement: Multimedia Appendix 1 [file mhealth_v9i9e31637_app1.pdf]

List of devices and infrastructure used for the measurements of heart rate and acceleration.

Measurements	Equipment model (Name of the	Sampling Frequency	Interval	Note
Physical workload				
Electrocardiogram (Sensing wear)	COCOMI (TOYOBO Co., Ltd.)	--	--	Stretchable conductive film
HR sensor	WHS-2 (Union Tool Co., Ltd.)	1 kHz	Per beat	Analysis of RRI
3-axis acceleration		31.25 Hz	Per beat	Capacitive sense
Infrastructure				
Data acquisition time	CC2650 & ThinkPad (Texas Instruments & Lenovo Co., Ltd.)	1 msec.	Per beat	Synchronized time with server
Data transfer	Raspberry Pi Zero W (Raspberry Pi Foundation)	--	--	IEEE802.11 b/g/n (Wireless LAN) Bluetooth 4.1
