# Supplementary material for: Psychological Effects of Heart Rate and Physical Vibration on the Operation of Construction Machines: Experimental Study
Source: JMIR Mhealth Uhealth. 2021 Sep 15;9(9):e31637. doi: 10.2196/31637 (PMC8482169; doi:10.2196/31637)
Supplement: Multimedia Appendix 2 [file mhealth_v9i9e31637_app2.pdf]

Supplementary Materials - Measurement data of the operator collected in the work environment.

Operation type	ID	driving time [sec]	LF/HF	LF nu	LF power [%]	RRI [msec.]	%HRR [%]	SDNN [msec]	RMSSD [msec]	MSE	Aw [m/s^2]	VDV [$m/s^{1.75}$]	MSDV [m/s^2]
A. riding operation	A-1	327	3.46	77.60	26.81	636.40	17.29	69.70	12.90	7.13	44.72	2.52	134.9
	A-2	288	6.01	76.20	32.24	591.50	12.61	71.50	19.60	5.12	59.88	2575.00	137.3
	A-3	404	2.91	74.42	27.14	558.90	11.79	30.90	14.40	4.63	67.89	15680.00	131.9
	A-4	301	3.63	78.39	35.83	742.20	5.12	46.60	18.50	7.34	68.50	5361.00	137.0
	A-5	348	7.61	88.39	41.12	647.90	6.43	35.10	19.90	6.84	54.61	6560.00	133.8
	A-6	378	6.32	86.34	32.70	601.20	15.30	93.90	21.30	6.49	69.91	3705.00	130.3
	A-7	271	4.23	81.04	11.62	619.10	21.62	64.20	20.40	8.89	38.34	3345.00	135.1
	A-8	258	2.63	59.70	30.47	792.10	9.95	83.30	23.30	6.77	84.73	6152.00	131.7
	A-9	238	3.11	74.70	32.55	753.60	12.08	103.80	22.10	6.10	22.67	7476.00	137.1
	A-10	363	5.54	84.14	25.04	680.40	12.10	77.40	26.80	8.30	90.70	8.52	134.9
	A-11	355	6.25	86.20	33.40	783.90	6.10	88.10	33.90	7.69	69.80	5100.00	130.5
	A-12	243	5.80	85.30	38.51	799.60	5.33	72.50	30.50	8.61	64.60	5098.00	130.2
	A-13	292	4.39	70.56	32.66	540.40	24.00	39.30	36.00	8.34	46.65	8324.00	133.9
	A-14	282	4.37	57.88	35.41	563.30	21.68	54.30	39.00	7.58	67.30	10110.00	136.2
B. remote operation by monitor	B-1	496	2.87	74.20	21.72	672.30	14.47	99.60	21.40	12.90	17.67	0.64	124.4
	B-2	493	1.29	56.40	38.03	727.60	2.90	88.40	23.30	13.51	45.52	4.20	130.3
	B-3	676	1.14	53.21	32.60	596.20	7.93	20.30	14.40	16.10	0.06	0.00	128.7
	B-4	446	1.20	54.61	30.25	824.10	0.62	31.00	13.30	13.00	28.80	4.20	130.8
	B-5	378	2.42	71.23	42.67	623.90	8.47	65.70	10.20	9.66	8.02	8.02	125.9
	B-6	482	1.42	58.67	40.47	746.60	4.29	34.30	25.10	10.50	0.01	0.85	127.7
	B-7	390	1.48	72.43	11.33	1006.40	1.06	30.60	23.60	15.10	13.00	1.23	125.0
	B-8	387	1.00	49.89	23.74	1015.50	0.76	41.80	26.50	12.74	6.26	0.60	125.9
	B-9	387	1.48	72.88	29.22	1027.40	0.39	63.90	18.50	12.63	15.80	1.51	127.0
	B-10	392	2.85	62.68	28.42	833.20	3.77	122.50	35.90	8.42	19.28	2.28	123.8
	B-11	404	3.03	75.20	36.85	885.60	1.57	120.90	46.90	9.15	12.25	0.44	123.7
	B-12	416	2.85	74.04	33.67	867.50	2.30	108.40	25.60	6.82	5.57	0.63	126.3
	B-13	387	1.85	64.94	30.32	893.40	1.47	93.10	35.90	9.51	15.80	0.55	126.8
	B-14	372	0.99	49.81	18.98	885.50	1.78	76.60	33.50	8.20	2.33	0.07	124.4
C. remote operation by VR	C-1	475	4.72	82.50	32.15	712.60	11.65	86.70	25.80	5.36	33.10	3.35	129.9
	C-2	463	4.22	80.80	30.14	530.50	18.58	53.80	14.70	7.43	58.35	4.69	128.2
	C-3	495	2.06	67.31	26.91	587.10	8.83	46.30	24.10	5.80	74.10	8647.00	135.4
	C-4	380	2.80	73.70	34.34	820.70	0.79	29.10	14.50	7.74	64.80	4.79	131.7
	C-5	358	3.34	77.05	32.76	671.60	4.55	65.60	17.20	5.18	88.80	8.95	130.0
	C-6	469	1.80	64.21	38.56	820.40	0.19	56.50	25.20	6.67	30.50	2880.00	130.9
	C-7	307	2.95	75.64	34.04	1014.60	0.79	31.50	25.60	5.57	47.10	4.38	129.8
	C-8	307	2.69	33.29	38.33	1027.50	0.38	32.80	27.10	8.14	53.80	5.10	128.7
	C-9	306	2.36	70.25	26.26	986.80	1.71	78.00	20.80	6.20	92.60	14.12	127.4
	C-10	375	3.84	84.71	32.83	749.90	7.89	36.80	33.20	10.60	60.60	6.07	134.5
	C-11	367	4.73	82.56	44.96	777.10	6.45	77.00	38.30	10.80	77.40	7.33	130.9
	C-12	251	4.49	81.79	24.79	753.80	7.68	71.30	34.70	8.43	47.40	4.48	133.2
	C-13	312	3.31	76.77	30.48	877.11	2.11	88.00	29.20	3.94	24.40	1.40	135.4
	C-14	312	2.49	71.37	30.79	912.10	0.76	87.20	39.40	4.45	38.40	3.61	131.6
Average		376.61	3.21	70.69	31.29	779.70	6.81	67.09	25.85	8.69	41.87	1776.29	130.05
SD		89.34	1.65	12.52	7.38	145.80	6.65	27.69	8.95	2.94	28.29	3068.39	3.75
