# Supplementary material for: Psychological Effects of Heart Rate and Physical Vibration on the Operation of Construction Machines: Experimental Study
Source: JMIR Mhealth Uhealth. 2021 Sep 15;9(9):e31637. doi: 10.2196/31637 (PMC8482169; doi:10.2196/31637)
Supplement: Multimedia Appendix 3 [file mhealth_v9i9e31637_app3.pdf]

Measurement indexes for work vibrations in operating work.

Measurement parameter	Method	Unit
Work vibration		
$a_w(t)$	$\sqrt{Kx^2 \cdot Acc(t)_x^{\ 2} + Ky^2 \cdot Acc(t)_y^{\ 2} + Kz^2 \cdot Acc(t)_z^{\ 2}}$	m/s^2
Aw	$\frac{1}{T}(\int_0^T a_w(t)^2 dt)^{1/2}$	m/s^2
VDV[a]	$(\int_0^T a_w(t)^4 dt)^{1/4}$	$m/s^{1.75}$
$MSDV_z$	$(\int_0^T a_{w_z}(t)^2 dt)^{1/2}$	m/s^2

[a]vibration doses value
