# Supplementary material for: Psychological Effects of Heart Rate and Physical Vibration on the Operation of Construction Machines: Experimental Study
Source: JMIR Mhealth Uhealth. 2021 Sep 15;9(9):e31637. doi: 10.2196/31637 (PMC8482169; doi:10.2196/31637)
Supplement: Multimedia Appendix 4 [file mhealth_v9i9e31637_app4.pdf]

The results of normality tests for HRV, physical workload, work vibration and machine operating.

Parameters	Kolmogorov-Smirnov		Shapiro-Wilk		Normality test result
	Statistic	*P*-value	Statistic	*P*-value	
**HRV time-domain**					
RRI	0.0768	.772	0.954	.0893	NP[a]
SDRR	0.0991	.377	0.959	.134	NP
RMSSD	0.107	.261	0.970	.322	NP
**HRV frequency-domain**					
LF_{nu}	0.1570	.0109	0.9264	.0098	P[b]
LF_{power}	0.1303	.0707	0.9468	.0498	NP
LF / HF	0.122	.122	0.9445	.0414	NP
MSE	0.1439	.0288	0.935	.0189	P
**Physical workload**					
%HRR	0.1424	.032	0.8860	<.001	P
**Work vibration**					
Aw	0.0969	.413	0.953	.0791	NP
VDV	0.388	<.001	0.6628	<.001	P
$MSDV_z$	0.0809	.699	0.962	.180	NP
**Machine operating**					
Running time	0.1242	.1059	0.9321	.0153	NP

[a]NP (non-parametric) performed multiple comparisons using the Steel-Dwass method in the Kruskal-Wallis test.

[b]P (parametric) performed multiple comparisons using the Bonferroni test.
